# Japanese Food Allergy-Labeling System and Comparison with the International Experience; Detection and Thresholds

**DOI:** 10.14252/foodsafetyfscj.D-21-00008

**Published:** 2021-12-07

**Authors:** Hiroshi Akiyama, Reiko Adachi

**Affiliations:** 1 Hoshi University, School of Pharmacy, Department of Analytical Chemistry, 2-4-41 Ebara, Shinagawa-ku, Tokyo 142-8501, Japan.; 2 National Institute of Health Sciences, Division of Foods, 3-25-26 Tonomachi, Kawasaki-ku, Kawasaki 210-0821, Japan.; 3 National Institute of Health Sciences, Division of Biochemistry, 3-25-26 Tonomachi, Kawasaki-ku, Kawasaki 210-0821, Japan.

**Keywords:** detection, labeling, food allergy, ELISA, risk assessment

## Abstract

In the Japanese allergy-labeling system, food labeling is mandated for 7 specific ingredients (egg, cow’s milk, wheat, buckwheat, peanut, shrimp, and crab) and recommended for 21 food ingredients in reference to case numbers of actual illness and the degree of seriousness. To monitor the validity of the labeling system, official methods for the detection of specific ingredient proteins in processed foods were developed. The official methods consist of ELISA methods for screening, and western blot methods for egg and milk, and PCR methods for wheat, buckwheat, peanut, shrimp/prawn, and crab as confirmation tests. The official methods consist of ELISA methods for screening, and western blot methods for egg and milk, and PCR methods for wheat, buckwheat, peanut, shrimp/prawn, and crab as confirmation tests. Threshold amounts (a few mg/kg) for labeling were set based on the approach of the analytical detections. Any foods containing protein allergens should be labeled if these contain allergens at greater than 10 ppm (mg/kg). Validation protocol criteria were established to standardize the Japanese official method. Food Safety Commission of Japan conducted a risk assessment of egg as a specific ingredient and judged that current labeling system for foods containing allergens is generally appropriate for “eggs”. In the future, it is important to accumulate necessary scientific knowledge in order to carry out food health impact assessment including further refinement. The Japanese experience and knowledge of food allergy-labeling system would contribute to harmonize international labeling guidelines to protect allergic consumers globally.

## 1. Introduction

The international Codex Alimentarius recommended the labeling of eight food ingredients (cereals containing gluten, crustaceans and products, eggs and egg products, fish and fish products, peanuts, soybeans and products, milk and milk products including lactose, tree nuts and nut products), known as the “Big 8”, in 1999^1^^)^.

In reference to a national survey of food allergy cases from 1997 to 1998^2^^,^^3^^)^ in Japan, a food labeling system for allergenic ingredients was mandated under the Food Sanitation Act of the Ministry of Health, Labour and Welfare (MHLW) on April 1, 2002. In 2010, management of the food labeling policy was transferred from the MHLW to the Japanese Consumer Affairs Agency (CAA). The CAA established the Food Labeling Act, which came into effect in 2015. In this act, food allergy-labeling is divided into two levels, namely, mandatory and recommended levels, according to the case numbers of actual illness and the degree of seriousness. Currently, egg, cow’s milk, wheat, buckwheat, peanuts, shrimp and crab require mandatory labeling as “specific ingredients” by Cabinet Office Ordinance. In addition, the notification recommends that foods containing ingredients such as abalone, almond, squid, salmon roe, orange, cashew nut, kiwi fruit, beef, walnut, sesame, salmon, mackerel, soybean, chicken, banana, pork, matsutake mushroom, peach, yam, apple, and gelatin be labeled as “sub-specific ingredients” (Table 1).

**Table 1.**
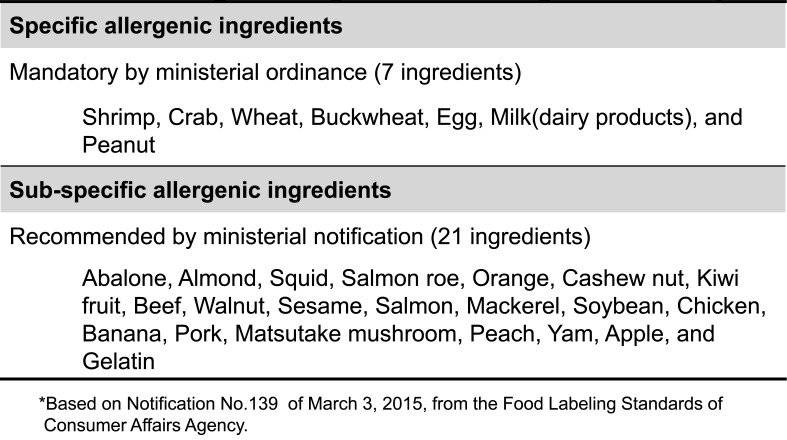
Allergenic ingredients designated in Japan*

In 2004, the MHLW revised the recommended labeling list to include banana, as the survey from 2001 to 2002 revealed an increase in the number of allergic patients. In 2008, the MHLW revised the mandatory labeling for shrimp and crab, since crustaceans have almost unlimited uses in processed foods in Japan. According to the 2004-2005 survey, crustaceans are a frequent cause of adverse food reactions in allergic patients. In 2013, the CAA revised the recommended labeling list to include cashew nuts and sesame due to the increase in number of allergic patients and because they were a frequent cause of adverse food reactions in allergic patients according to the 2011-2014 survey. In 2019, the CAA revised the recommended labeling list to include almond due to the increase in number of patients with almond allergy and because they were a frequent cause of adverse food reactions in allergic patients according to the 2016-2017 survey. To our knowledge, Japan is one of the first countries to set up a mandatory food allergy-labeling system and regulate it under a national act.

## 2. Japanese Allergenic Ingredient Labeling System^4^^)^

In Japan, in principle, the names of specific ingredients, etc. in the Food Labeling Standards must be used; however, alternative labels are also specified. Examples of alternative labels are eggs, chicken eggs, duck eggs, quail eggs, and alike. Meanwhile, labeling of combine specific ingredients, etc. is not allowed. For example, complex labeling such as wheat and soybean as “cereals” or beef, pork, and chicken labeled as “meat” or “animal XX” is not allowed.

However, in cases of five ingredients— (1) protein hydrolysate, (2) fish sauce, (3) ground fish meat, (4) fish oil, and (5) seafood extract— these food products consist of seafoods caught indiscriminately with fishing net. Since it is not possible to know whether specific fish and shellfish are contained in the product, complex labeling method is allowed for exception, such as display of “including seafood”.

For additives derived from specific ingredients, the description “food additives” and the fact that the additives are derived from specific ingredients will be displayed. In addition, for foods containing additives derived from specific ingredients, it is indicated that the additives are contained and that these additives are derived from the specific ingredients, such as “additive name (derived from XX)”.

In Japanese food allergy-labeling system, precautionary allergen labeling (PAL) of “may be included” is prohibited. While PAL is accepted in western countries, it differs from the Japanese labeling system, which allows warning declaration outside the label margin. For example, declarations are recommended when contamination occurs by using the same factory production line, “this facility manufactures products containing XX (name of specific ingredient, etc.)”, “YY (name of specific ingredient, etc.)”, “manufactured with the same equipment used specific ingredient.” etc., and when contaminated by the collection method of ingredients, “the small fish used in this product is caught by a fishing method in which XX (name of specific ingredient, etc.) is mixed”. However, this warning declaration is not considered labeling, because the declaration is written outside the margin.

**Fig.1.**
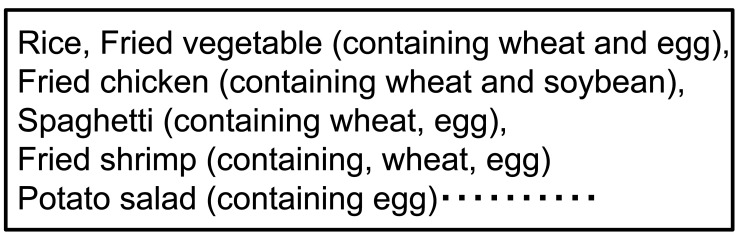
Example of individual allergen declaration

Fig. 1 shows examples of individual allergen declarations on a prepackaged lunch at a shop. Under the current Japanese Labeling Act, all allergens must be individually labeled, except for certain cases. This provides information needed for consumers to select the appropriate item.

**Fig.2.**
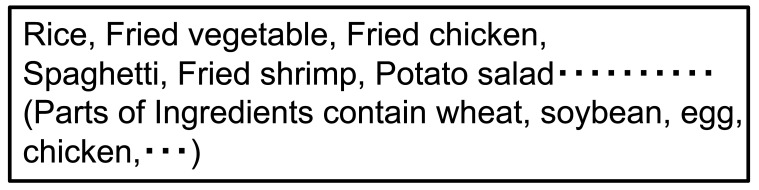
Example of collective allergen declaration

Fig. 2 shows an example of collective allergen declaration on a prepackaged lunch at a shop. Consumers can see the list of declared allergens on labeling. If allergens are to be listed collectively, all allergens must be declared at the end of ingredient list.

## 3. Japanese allergy-labeling thresholds and detection methods for specific ingredients

Food allergy-labeling system is necessary for people with allergies. However, in general, the proteins and nucleotides in specific ingredients are not necessarily hazardous substances. The threshold dose for an allergic reaction is often considered to be zero or below the limit of detection (LOD). However, zero tolerance of offending foods would create enormous practical problems for the food industry. Therefore, the MHLW established a food allergy-labeling threshold and developed official detection methods for specific ingredients.

To this end, a detection method study group, consisting of manufacturing companies, retailers, public research institutes, universities, and private inspection institutes, was organized in 2001. As a result, detection methods for specific ingredients in foods were developed to monitor the validity of labeling.

**Fig. 3.**
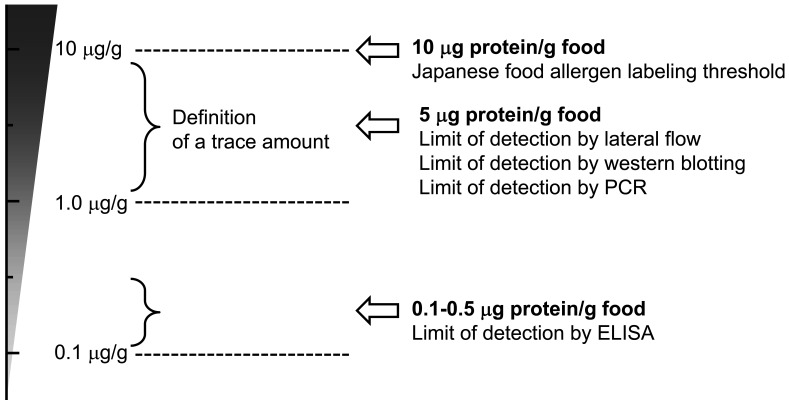
Consideration of Japanese food allergen-labeling threshold

The detection method study group considered how to set the threshold for labeling (Fig. 3). The group presumed that the LOD for enzyme-linked immunosorbent assay (ELISA) is generally in the range of 0.1-1.0 μg protein/g food. However, setting labeling threshold in the range of the LOD of ELISA would be very challenging because of the large deviations among laboratories for repeatability and reproducibility. In addition, the LODs of the lateral flow and polymerase chain reaction (PCR) methods are approximately 5 μg protein/g food^3^^,^^5^^)^.

Meanwhile, another labeling study group, consisting of medical doctors, government officials, patient representatives, manufacturing company representatives, and public health researchers, determined the approximate threshold for the labeling system as the definition of a trace amount. The group stated, “if more than a few micrograms of protein weight per milliliter of food or a few micrograms of protein per gram of food are contained in a food, labeling of that allergen is necessary according to the experience of clinical standpoints.”

Considering these factors, the MHLW designated 10 μg protein/g food (the corresponding allergen soluble protein weight/food weight) as the threshold for monitoring of labeling using analytical methods such as ELISAs. MHLW considered that this level is the minimum value for controlling contamination of specific ingredients using detection methods on an industrial scale.

Therefore, the MHLW attempted to develop detection methods over presence of proteins at the level of a few micrograms per milliliter or gram of foods, based on the definition of a trace amount.

However, accurate determination of specific protein ingredients is difficult since proteins can undergo structural changes as a result of denaturation and degradation. Furthermore, specific ingredient protein reference standards could change because calibrator proteins in each ELISA method cannot always be obtained for every test. In Japan, the labeling of egg, milk, wheat, buckwheat, and peanut in any processed foods became mandatory in April 2002, followed by shrimp and crab joining this mandatory list in June 2008. The Japanese official methods consisted of two different ELISA kits for screening, the western blot method for egg or milk and the PCR method for wheat, buckwheat, peanut, shrimp/prawn, and crab as confirmation tests under the ministerial notification^3^^,^^5^^)^. MHLW included the specification and standardization of the extraction buffer, reference material, and the standard solution for the testing of these five specific allergens in 2004. Furthermore, the validation protocol criteria were included in the official guidelines of 2006 to standardize the Japanese official method for allergen detection^3^^,^^5^^,^^6^^)^, followed by addition of ELISA and PCR methods, reference material, and the standard solution for testing of crustaceans for detection of shrimp/prawn and crab in 2008. Validation protocol criteria to evaluate the equivalency between established methods and those with minor improvements were added to the official guidelines in 2010 with the replacement of reducing reagent 2-mercaptoethanol (2ME) with sodium sulfite in the extraction buffer^5^^,^^7^^)^, followed by improvements in the standard solution and calibrators.

## 4. ELISA Methods

ELISA is the most commonly used method in the food industry and official food-control agency’s laboratories for detecting and quantifying trace amounts of specific ingredients in foods. Two ELISA-based assay kits were introduced as the Japanese official methods in 2002^3^^,^^5^^,^^6^^)^. The optimal antibody for detecting specific ingredient proteins in foods was previously determined. Antibodies can be classified as either monoclonal or polyclonal. At this time, a polyclonal antibody was chosen for detecting a variety of specific ingredient proteins since the structure of the allergen would be denaturated by food processing. For ELISAs, the target proteins can be divided into 2 types: whole proteins and proteins specific certain ingredients.

One of the kits for the five specific ingredients (egg, milk, wheat, buckwheat, peanut, and soybean) is the FASTKIT ELISA series (Food Allergen Screening Test Kit). This kit is produced commercially by NH Foods, Ltd., and uses polyclonal antibodies against multiple antigens to detect whole allergen proteins. Basically, many specific ingredients contain multiple particular proteins, such as eggs contain ovalbumin, ovomucoid, and lysozyme. These proteins can be denatured, degraded, and combined with other proteins via food processing. To solve this problem, this kit adopts multiple antibodies for the native protein, in addition to antibodies for the denatured proteins. Currently, the FASTKIT ELISA Ver. III^®^ series for each specific ingredient has been commercialized following an equivalency test to the FASTKIT ELISA Ver. II^®^ series^3^^,^^5^^,^^6^^)^.

Another ELISA kit for these five specific ingredients is the FASPEK KIT^®^, produced commercially by Morinaga Institute of Biological Sciences, Inc. This kit uses polyclonal antibodies to detect specific purified proteins or individual proteins of specific ingredients. In the FASPEK KIT^®^, these specific proteins are used as the target proteins. The target proteins are ovalbumin for egg, casein for milk, gliadin for wheat, the main allergen protein complex for buckwheat, and the protein complex including Ara h2 for peanut. At present, the FASPEK ELISA II^®^ series for ovalbumin, casein, β-lactoglobulin, gliadin, buckwheat main allergen protein complex, peanut allergen protein complex including Ara h2, and soybean have been commercialized after an equivalency tests to the FASPEK ELISA^®^ series^3^^,^^5^^,^^6^^)^. The ovalbumin kit for egg and the casein kit for milk are used as Japanese official methods, because of the high proportions of these proteins present in egg and milk.

In 2010, the addition of ALLERGENEYE^®^ ELISA series of kits for egg, milk, wheat, buckwheat and peanut as Japanese official methods was announced following validation by the Japanese validation protocol. This kit is produced commercially by Prima Meat Packers, Ltd., and uses monoclonal antibodies to detect specific purified proteins or individual proteins of specific ingredients. The target proteins are ovalbumin for egg, β-lactoglobulin for milk, gliadin for wheat, the 24 kDa protein for buckwheat, and the Ara h1 protein for peanut. At present, the ALLERGENEYE^®^ ELISA II series for ovalbumin, β-lactoglobulin, gliadin, 24-kDa protein, Ara h1 protein, and soybean have been commercialized following an equivalency tests to the ALLERGENEYE^®^ ELISA series^3^^,^^5^^,^^6^^)^.

Following the designation of shrimp/prawn and crab for mandatory labeling in June 2008, two ELISA methods were developed for determination of crustacean proteins in processed foods^3^^,^^5^^,^^6^^)^; FA test EIA-Crustacean [Nissui^®^] produced by Nissui Pharmaceutical Co., Ltd.,^8^^)^ and Crustacean Kit [Maruha Nichiro^®^] produced by Maruha Nichiro Corporation^9^^)^. Both kits use tropomyosin as the target protein for black tiger prawn, and have been validated according to the Japanese validation protocol^3^^,^^5^^,^^6^^)^. At present, FA test EIA-Crustacean II [Nissui^®^]^8^^)^ and Crustacean Kit II [Maruha Nichiro^®^]^9^^)^ have been commercialized after confirmation of equivalency to the FA tests EIA-Crustacean [Nissui^®^] and Crustacean Kit [Maruha Nichiro^®^], respectively. The commercial ELISA kits are shown in Table 2.

**Table 2.**
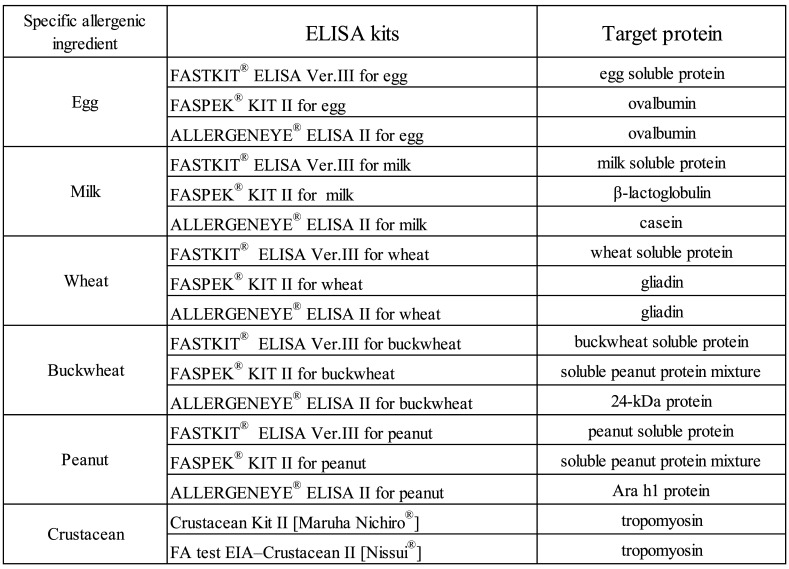
Commercial ELISA kits for specific allergenic ingredients

Detection with consistent sensitivity of every kind of protein within a foodstuff is impossible by using one kind of ELISA system, as contents and denaturation of proteins vary greatly. Determination by ELISA is affected by the denaturation and extraction efficiency of the target protein. Conventional methods cannot be easily applied to highly heated and pressure-processed foods, e.g., retort and canned foods. Therefore, we developed a unique buffer for extracting insoluble antigens produced during heat and pressure processing^3^^,^^5^^)^ as well as novel polyclonal antibodies for the extracted protein allergens using the specific extraction buffer (including detergent and reducing reagent) in the Japanese official method kits.

## 5. Reference Materials and Calibrators^3^^,^^5^^,^^10^^)^

To assess compliance to the mandatory labeling system in processed foods sold in Japan for specific ingredients (egg, milk, wheat, buckwheat, and peanut), established in April 2002 (shrimp and crab added in June 2008), monitoring using the two types of ELISA by local government and health centers began in 2002. However, as the regulation came into effect, some discrepancies became apparent between results obtained from two kits, partly due to difference in antibodies used. The discrepancies could also have been due to the different standard solutions provided in the kits. Since the test kits are used for regulatory purposes, the authority considered that the extraction buffer and reference standard for measurement should be harmonized and standardized between the test kits. Therefore, ministry authorities set the specifications and standardization for the extraction buffer, which included SDS as detergent and 2ME as reducing reagent, reference material and standard solution for testing of five specific ingredients in 2004^3^^,^^5^^)^. In 2008, 2ME was classified as a “hazardous” material by the Globally Harmonized System of Classification and Labeling of Chemicals, claiming that it posed an ecological burden when disposed and its unpleasant odor meant that it had to be handled in a chemical fume hood. Furthermore, following amendment of the Poisonous and Deleterious Substances Control Act of Japan in 2008, 2ME was designated as a poisonous substance, necessitating strict handling. The replacement of 2ME with sodium sulfite was included in the specifications and standardization^7^^)^.

Specifications and standardization include ingredients, preparation method of the standard solution, concentration of proteins, and the main band on SDS-PAGE, as shown in homepage of CAA^3^^,^^5^^)^. Table 3 shows the ingredients and the preparation method of the initial extracts. To prepare the calibrators, ingredients are extracted with the standard solution containing SDS and sodium sulfite. The initial extract is prepared by centrifugation and filtration of the extract. The diluted extract is then prepared by 10-fold dilution of the initial extract with phosphate-buffered saline (PBS) (pH 7.4). Protein concentration of the diluted extract is assayed by using the 2-D Quant kit (GE Healthcare Bio-Sciences Co). The standard solution is then prepared by a 2-fold dilution with PBS (pH 7.4) containing 0.2% BSA. The calibrator included in each commercial kit is prepared by dilution of the standards (concentrated standard solution) to 50 ng/mL with the original buffer of each company’s kit containing the carrier protein.

**Table 3.**
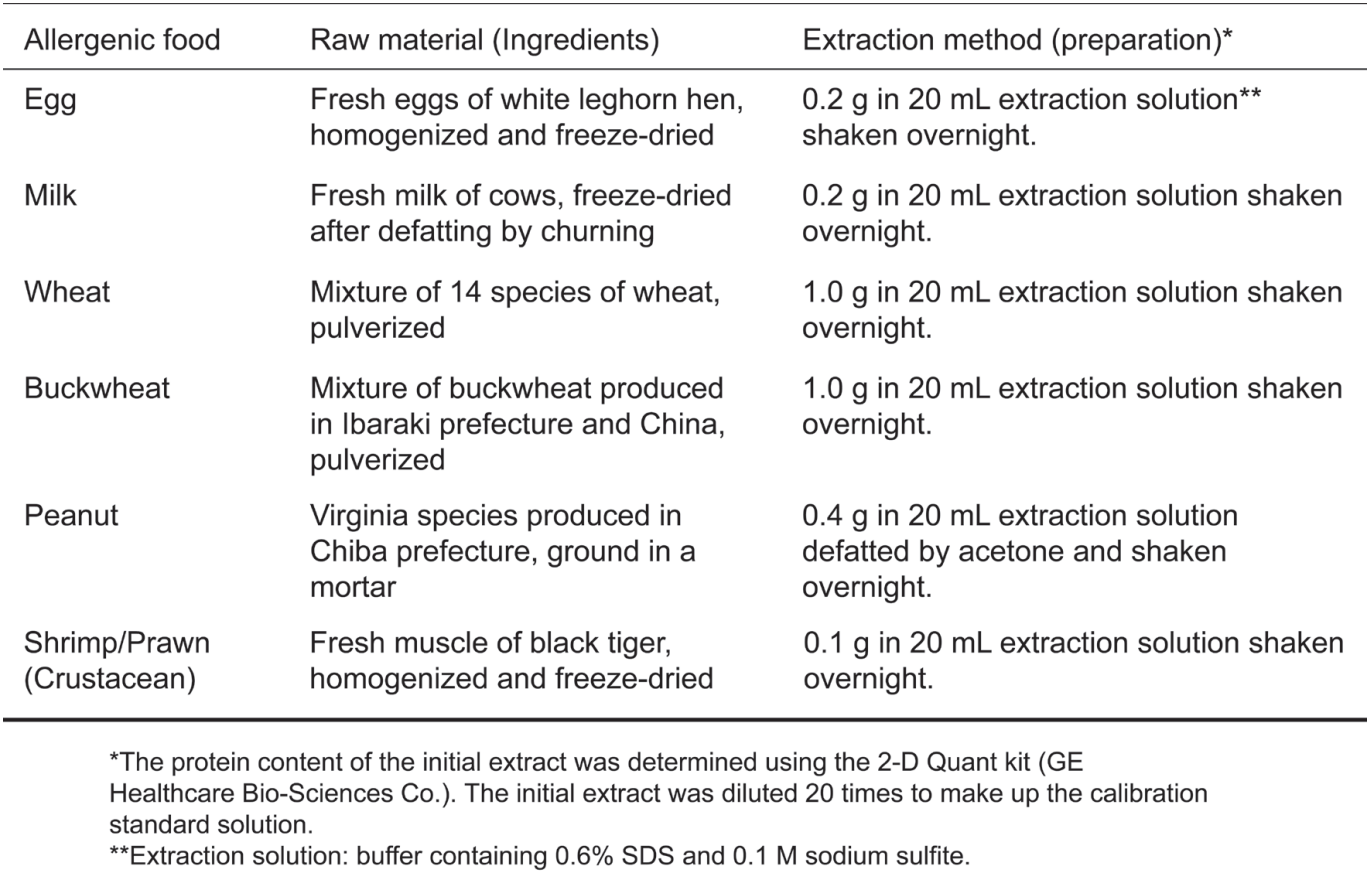
Raw materials and initial extraction methods

**Fig. 4.**
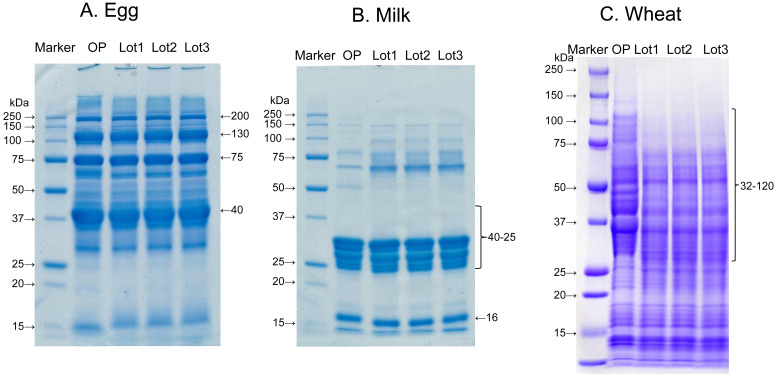
SDS-PAGE images of standard proteins of egg, milk and wheat. OP: original powder, Lot 1: sample lot 1, Lot 2: sample lot 2, Lot 3: sample lot 3

**Fig. 5.**
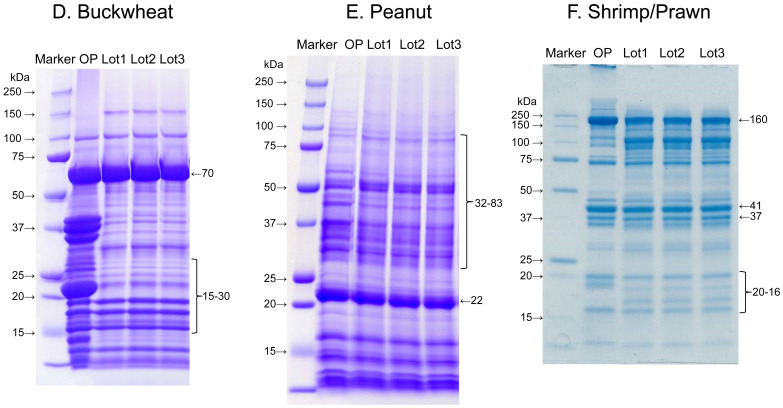
SDS-PAGE images of standard proteins of buckwheat, peanut and shrimp/prawn OP: original powder, Lot 1: sample lot 1, Lot 2: sample lot 2, Lot 3: sample lot 3

Three lots of initial extracts for each specific ingredient were prepared according to this procedure to assess conformation to the specifications. Reproducibility of the protein concentration and SDS-PAGE pattern of the initial extract solutions were also confirmed (Figs. 4 and 5).

The initial extract solutions were stored at -80°C for 6 months to evaluate their stability. Protein concentration and SDS-PAGE pattern of the 3 lots were equivalent and no significant variability occurred during the storage period. The calibration standard solution was stored at 4 and 37°C. The calibration standard solution was tested using the relevant ELISA kits once a month during storage, and the stability was confirmed by absorbance measurements.

## 6. Japanese Guideline Criteria for the Validation of Specific Ingredient Detection Methods^3^^,^^5^^)^

Full validation protocol criteria^10^^,^^11^^)^ were described in the 2006 official guidelines to standardization and endorsement as Japanese official methods for specific allergen detection. The validation protocol criteria of quantitative and qualitative allergen detection methods are outlined in Tables 4 and 5, respectively. If further developed detection method were to satisfy the validation protocol criteria, the method would be endorsed as the Japanese official method.

**Table 4.**
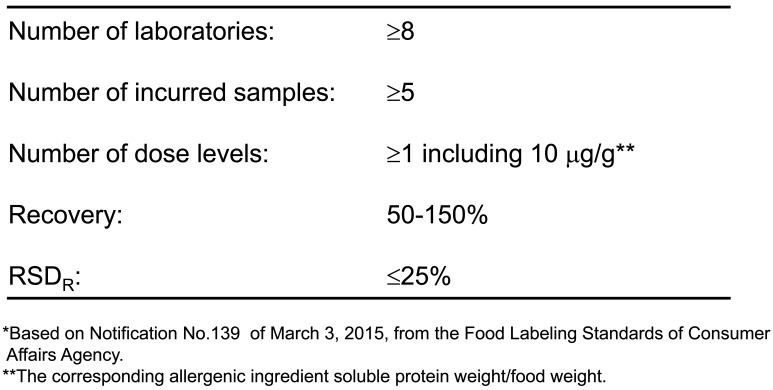
Japanese guideline criteria for validation protocol of quantitative detection methods for food allergenic ingredients*

**Table 5.**
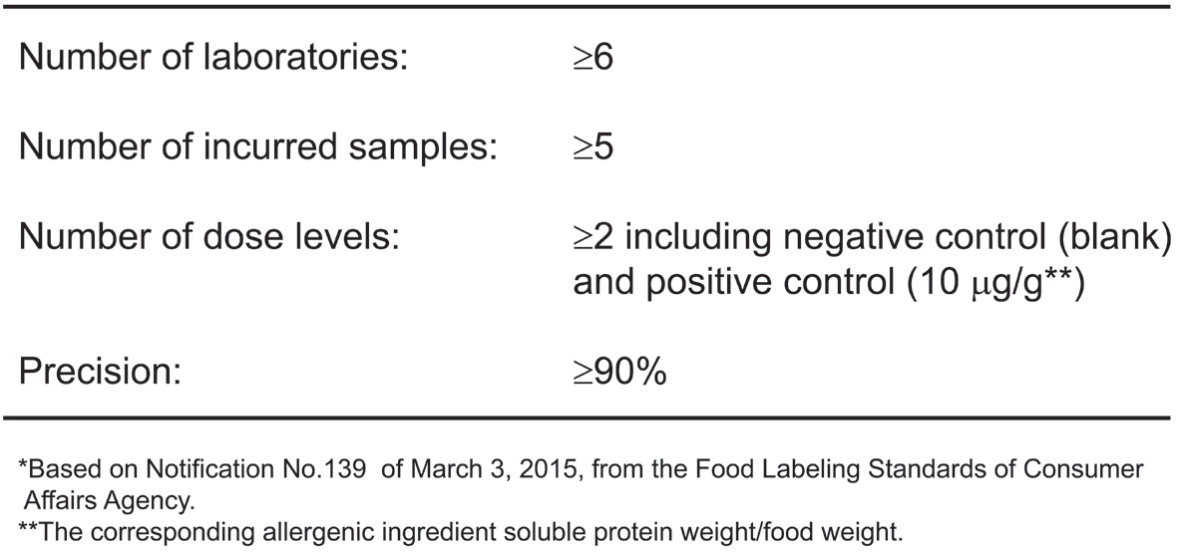
Japanese guideline criteria for validation protocol of qualitative detection methods for food allergenic ingredients*

The validation protocol criteria^10^^,^^11^^)^ for specific ingredient protein by quantitative detection methods are as follows: (1) eight or more laboratories (independent from the ELISA developer), (2) five or more food samples (matrices), and (3) a concentration of 10 μg/g food-specific ingredient in the food sample (the corresponding specific ingredient soluble protein weight/food weight), where the concentration is defined as “trace amount of contamination”, meaning any food containing the specific ingredient protein at greater than 10 μg/g must be labeled for the relevant specific ingredients under the Food Labeling Act. If the specific ingredient protein level is less than 10 μg/g, labeling is not required. The food sample should be prepared by common processing methods, such as heating, baking, frying, acidification, and pressurization processes, hereinafter termed “model processed (incurred) food”. It is recommended that food samples comprising animal or plant products, highly processed foods (long-term heating, high-pressure preparation), or acidic foods be evaluated during validation to ensure that the ELISA method is applicable to various types of processed foods, (4) the recovery rate from the model processed food should be in the range of 50 to 150%, and the inter-laboratory precision (RSD_R_) should be less than 25%, (5) the matrix effect data, by adding the target specific ingredient protein to the matrix extract, that of foods showing a false positive (cross-reactivity) or false-negative result, and that of matrices for which the ELISA method hardly applies should be fully examined and disclosed, (6) “Reference Material for Monitoring Foods Containing Specific Ingredients” should be applied for preparing kit standards as well as model processed food samples^10^^,^^11^^)^.

In the guidelines and reference materials, the initial extract solution and the extraction procedure for specific allergens are also indicated and standardized. In developing a food-specific allergen ELISA, the ELISA performance should fulfill the following inter-laboratory validation criteria of the “Collaborative Study” protocol based on ISO5725 (JIS Z8402), which is basically the same as that of AOAC^10^^,^^11^^)^, and the obtained performance data must be open for public.

## 7. Validation Study and Equivalency Study for ELISAs^6^^,^^10^^,^^11^^)^

Collaborative studies using each ELISA method with model processed foods containing specific ingredient proteins were performed. The five or six model processed foodstuffs were spiked with specific ingredients to a final amount of 10 μg/g in the ingredient stage^6^^,^^10^^,^^11^^)^. The use of model processed foods is considered to be the best way to assess established ELISA methods by inter-laboratory validation. First, a homogeneity test was conducted for the model processed foods. Basically, the procedure was performed following the AOAC homogeneity test protocol with some modifications.

Table 6 shows the inter-laboratory validation method. The first step involves the preparation of a standard curve (4-parameter logistic curve) using absorbance values collected from each participating laboratory. Second, the first and second sets of data are subjected to a repeatability test by using the average values from three wells. Third, Cochran’s test and Grubbs’s test are used to remove outliers (both tests were performed at a significance level of 5%). The final step involved performing a one-way analysis of variance (ANOVA). The ten participating laboratories included manufacturing companies, public research institutes, local public inspection institutes, and private inspection institutes. Tables 7–12 for the FASTKIT^®^ ELISA Ver. II series, the FASPEK^®^ ELISA series, the ALLERGENEYE^®^ ELISA series, the FA test EIA-Crustacean [Nissui^®^], and the Crustacean Kit [Maruha Nichiro^®^] show the validation results for egg, milk, wheat, buckwheat, peanut, and shrimp/prawn (crustacean), respectively^10^^,^^12^^)^.

**Table 6.**
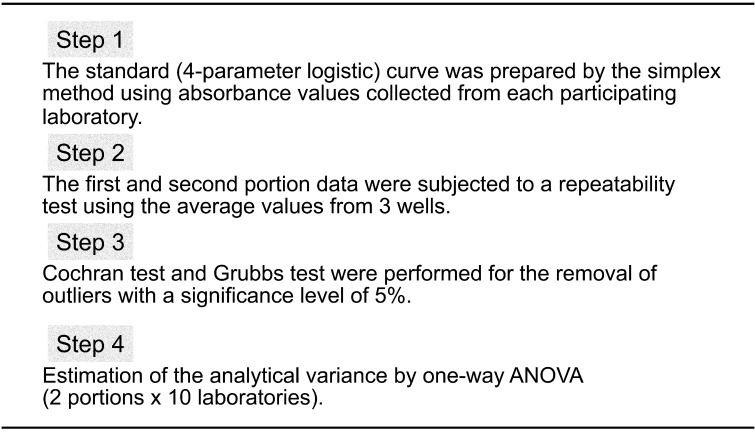
Evaluation method for the inter-laboratory study

**Table 7.**
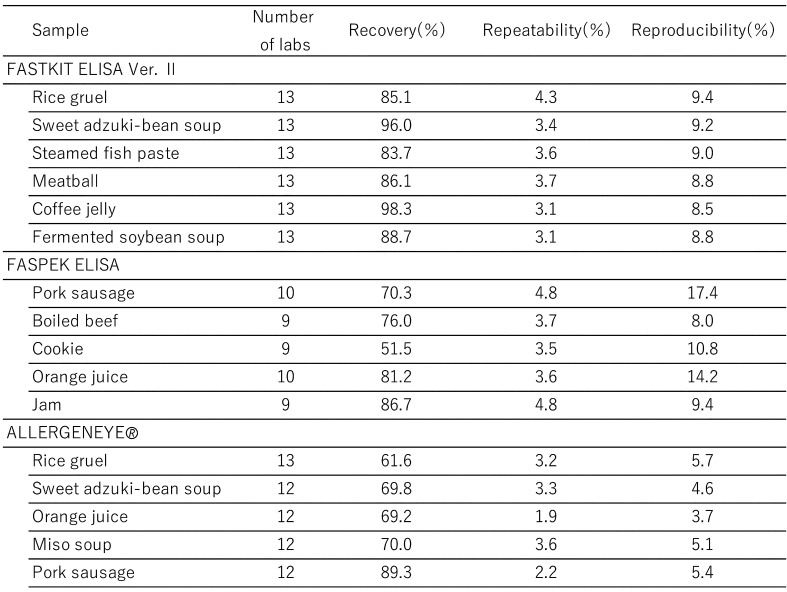
Recovery, repeatability and reproducibility for egg detection

**Table 8.**
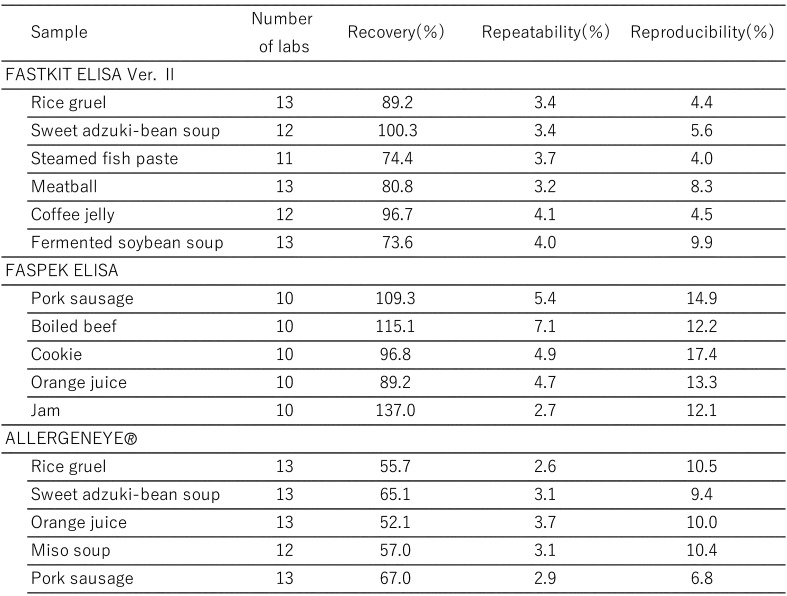
Recovery, repeatability and reproducibility for milk detection

**Table 9.**
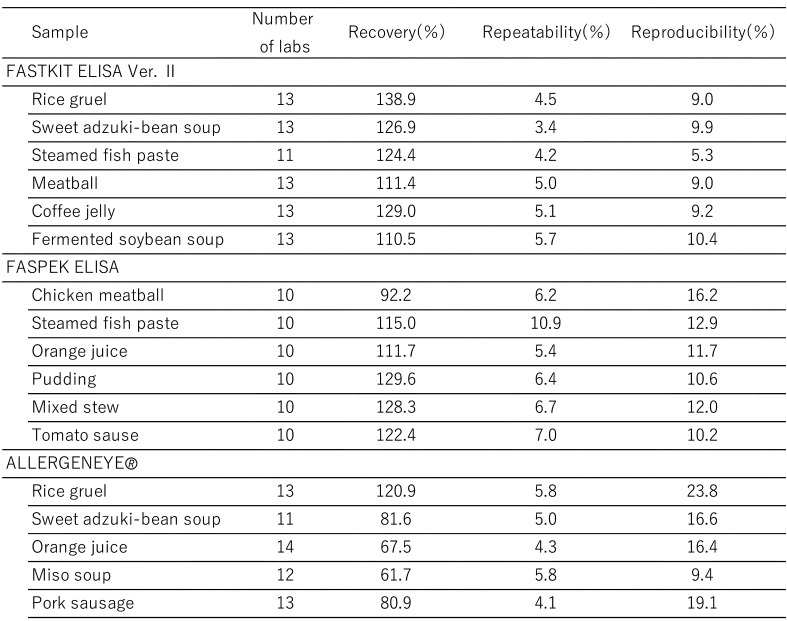
Recovery, repeatability and reproducibility for wheat detection

**Table 10.**
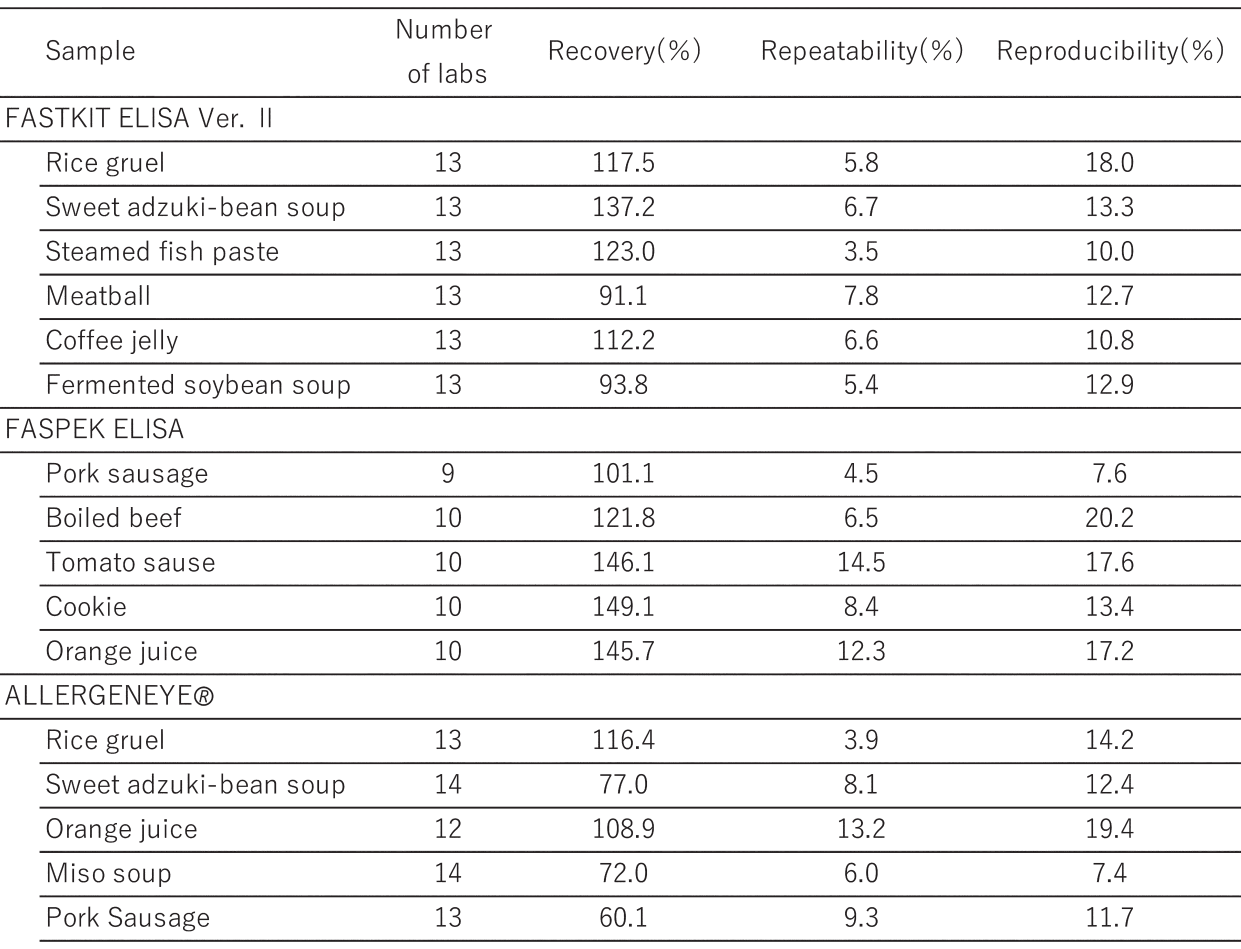
Recovery, repeatability and reproducibility for buckwheat detection

**Table 11.**
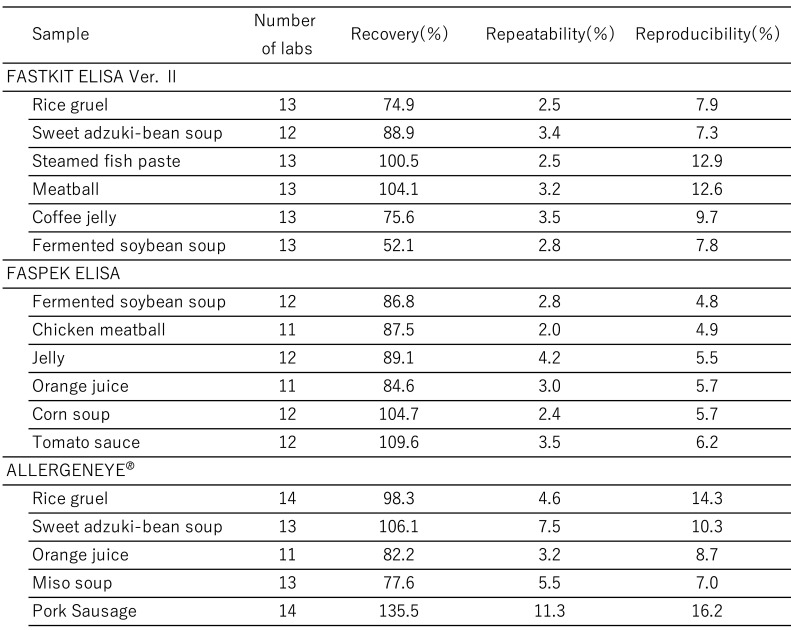
Recovery, repeatability and reproducibility for peanut detection

**Table 12.**
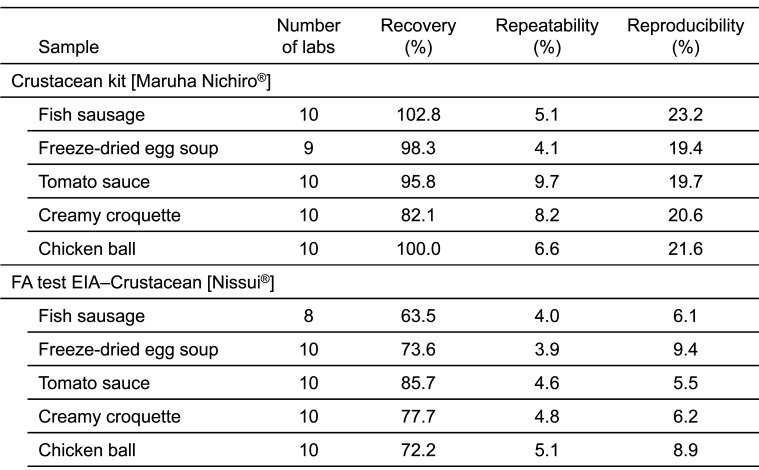
Recovery, repeatability and reproducibility for shrimp/prawn detection

These results were evaluated according to the AOAC protocol and ISO 5725-5 robust statistics. All kits meet the Japanese acceptance criteria.

Furthermore, following replacement of 2ME with sodium sulfite as a reducing reagent, an equivalency study between the current kit using 2ME and the improved kit using sodium sulfite for each ELISA method was conducted by specific ingredient protein determination of various food items. For equivalency studies between the FASTKIT^®^ ELISA Ver. II series and the FASTKIT^®^ ELISA Ver. III series, the correlation formulae were y=0.9139x (r^2^=0.9946) for egg, y=1.0614x (r^2^=0.9884) for milk, y=1.0466x (r^2^=0.9949) for wheat, y=0.8583x (r^2^=0.9977) for buckwheat, and y=0.9075x (r^2^=0.9834) for peanut. For equivalency studies between the FASPEK^®^ ELISA series and the FASPEK^®^ ELISA II series, the correlation formulas were y=0.9404x (r^2^=0.999) for egg, y=1.0399x (r^2^=0.995) for milk, y=1.086x (r^2^=0.999) for wheat, y=1.0767x (r^2^=0.994) for buckwheat, and y=0.8921x (r^2^=1.000) for peanut. For equivalency studies between the ALLERGENEYE^®^ ELISA series and the FASPEK^®^ ELISA II series, the correlation formulae were y=1.0114x (r^2^=0.9803) for egg, y=0.9437x (r^2^=0.998) for milk, y=0.9681x (r^2^=0.907) for wheat, y=1.1872x (r^2^=0.987) for buckwheat, and y=1.1885x (r^2^=0.990) for peanut. For the equivalency study between FA test EIA-Crustacean [Nissui^®^] and FA test EIA-Crustacean [Nissui^®^] II, the correlation formula was y=1.0621x (r^2^=0.977) for shrimp/prawn. For the equivalency study between Crustacean Kit [Maruha Nichiro^®^] and Crustacean Kit II [Maruha Nichiro^®^], the correlation formula was y=0.928x (r^2^=0.995) for shrimp/prawn.

## 8. Western Blotting Method^2^^,^^3^^,^^5^^)^

Western blotting is another protein-based qualitative method. Since DNA of egg and milk is naturally contained in chicken meat and calf meat, respectively, the specific detection of egg or milk by PCR method in processed foods is difficult. Therefore, western blotting method was adopted as a confirmation test for protein-based qualitative methods. This method has high specificity, because specific proteins are separated according to their molecular masses, irrespective of their original electrochemical charge. Samples are prepared for polyacrylamide gel electrophoresis (PAGE), and then subjected to blotting and blocking on the highly hydrophobic membrane. Next, the protein on the membrane is reacted with the primary antibody, followed by the secondary antibody, and then reacted with the avidin-labeled alkaline phosphatase-biotin conjugate, followed by the substrate. The final step is detection of the protein-derived allergens. Western blotting is prescribed as the confirmation test for egg and milk in the Japanese official methods, and western blotting kits for egg and milk are commercially available.

## 9. PCR Method

PCR is a DNA-based method that can specifically and sensitively detect allergenic ingredients in processed foods. PCR method was adopted as the confirmation test for wheat, buckwheat, and peanut in the Japanese official methods in 2002. Three DNA extraction methods (silica-membrane column-type kit, anion-exchange column-type kit, and the CTAB method) are prescribed in the Japanese official methods. The PCR target genes for detection of wheat^13^^)^, buckwheat^14^^)^, and peanut^15^^)^ are shown in Table 13. Primer pairs were designed to detect these gene sequences. To confirm the validity of the extracted DNA for PCR quality, primers recognizing the non-coding region of the chloroplast DNA were designed as an analytical control^3^^,^^5^^)^. To avoid false-negative results, it is important to check the validity of the extracted DNA for PCR.

**Table 13.**
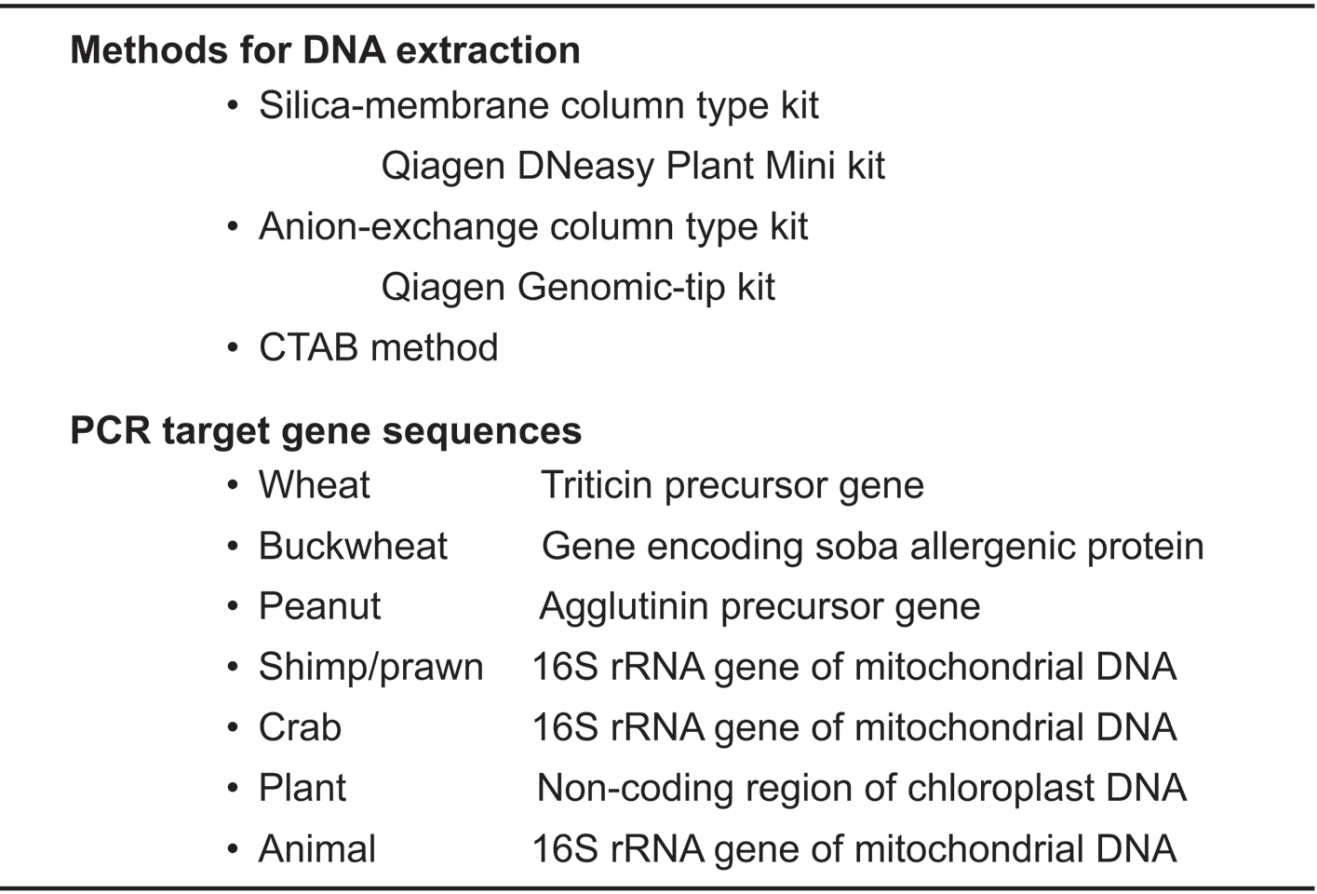
PCR method for wheat, buckwheat, peanut, shrimp and crab

Since mandatory labeling of shrimp/prawn and crab was designated in June 2008, PCR methods to discriminate between shrimp/prawn and crab in processed foods have been developed. Notably, the ELISA method for crustaceans cannot discriminate between shrimp/prawn and crab because of similar protein sequence homologies^16^^)^. Both methods have been validated according to the Japanese validation protocol^3^^,^^5^^,^^10^^)^ and both primers are commercially available.

Furthermore, PCR or real-time PCR methods for soybean, walnut, kiwifruit, banana, pork, chicken, and beef were developed^12^^,^^17^^,^^18^^,^^19^^,^^20^^,^^21^^)^. They are all on the recommended labeling list as sub-specific ingredients.

## 10. Immunochromatography Methods (Lateral Flow Method)

Immunochromatography methods (lateral flow method) using animal antibodies have also been developed. There are commercially available kits for seven items: egg, milk, wheat, buckwheat, peanut, crustacean, and soybean. Analysis is initiated by dropping the protein solution extracted from a food onto a test strip, and a target band is detected after 10 to 20 minutes if antigen-antibody reaction turns positive, which is similar to ELISA and Western blotting methods. This method does not require expensive equipment/materials and can be visualized easily and quickly^22^^,^^23^^,^^24^^)^. However, false-negatives are possible due to insufficient sensitivity, the influence of contaminants, and the hook effect or prozone effect (which occurs when excessive amounts of target protein are present).

## 11. LC-MS/MS Methods

Internationally, there have been many reports on the development of analytical methods for allergens in foods using LC-MS/MS. In Japan, Nagai et al developed a method for analyzing buckwheat allergens using LC-MS/MS^25^^)^. Seki et al developed an LC-MS/MS analysis method targeting peptides derived from trypsin digestion, i.e., glutenin of wheat including wheat, rye, barley, and oats and 13S globulin of buckwheat^26^^)^. The LC-MS/MS method enables specific and simultaneous detection of a plurality of allergens, and is an effective confirmation test, as with real-time PCR.

## 12. Practical Monitoring of the Allergy-labeling System^3^^,^^4^^,^^5^^)^

**Fig. 6.**
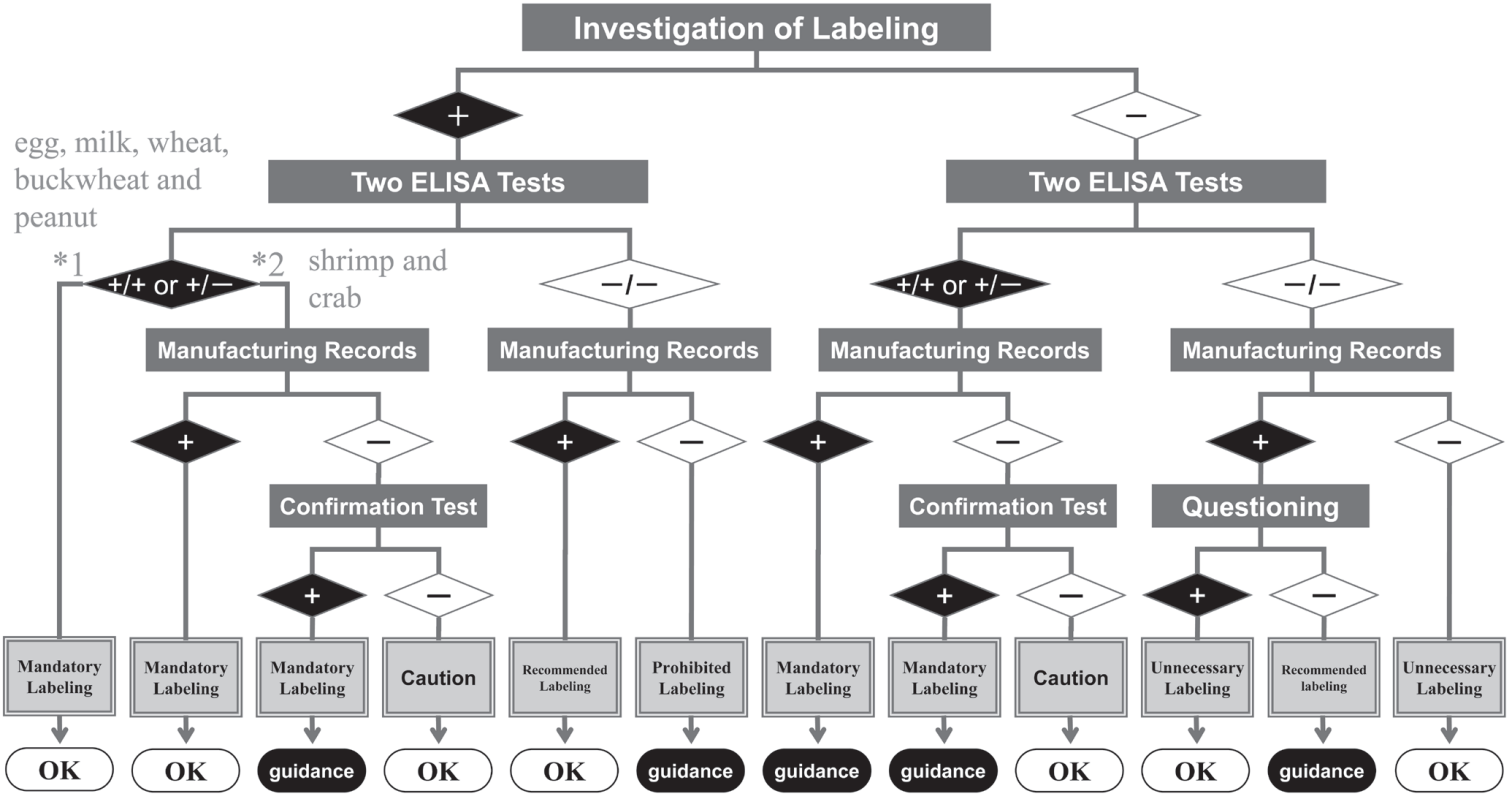
Decision tree for the practical test used to monitor the allergy-labeling system

Following is the outline for practical monitoring of the allergy-labeling system at a local government health inspection center. First, the local government investigates the food allergy-labeling. As a screening test, quantitative analyses using the two different official ELISA kits for specific ingredient protein are performed to double-check each specific ingredient. The authority determines the threshold for a positive value to be 10 μg/g in the screening test, according to the definition of a trace amount. Next, the authority examines the manufacturing records as a nonscientific verification. If the presence of the specific ingredient still cannot be determined, a confirmation test using western blotting for egg or milk or PCR for wheat, buckwheat, peanut, shrimp/prawn, or crab is performed. If an allergen is positive by the confirmation test, the labeling should be corrected according to the local government guidance. If a company does not follow the guidelines properly, it can be penalized under the Japanese Food Labeling Act. Fig. 6 shows the decision tree for practical monitoring of the allergy-labeling system. Local governments and health centers monitor labeling according to this decision tree. There have been cases of incorrect labeling of specific ingredients on processed food products, and such errors should be corrected using local government guidance.

## 13. Issues of Food Allergy-labeling in Japan

The food allergy-labeling system has been widely implemented in Japan, thus consumers can relatively, easily avoid specific ingredients by referring to product labelings. However, in recent years, research has demonstrated that the risk of developing food allergies in infants would rise with longer duration of food avoidance. Avoiding specific ingredients is a strategy used by those who have already developed food allergies to the ingredients; however, avoiding specific ingredients before an allergy onset increases the risk of allergy development in infants, especially in case of skin lesions (eczema/atopic dermatitis). The risk of allergy development in infancy is known to be high. If the foods, considered to be high-risk according to food-specific risk assessment, are avoided as a preventative measure, the risk of allergy development is thought to increase^32^**^)^**. Therefore, in recent years, the recommended diet for those with food allergies has been changed to the minimum necessary dietary guidance^27^^)^.

It is recommended that the minimum necessary dietary avoidance be continued for patients who can eat a small amount of allergenic protein even if they have not acquired tolerance, since the amount of allergenic protein in processed foods cannot be known. Therefore, if the protein content of specific ingredients in processed foods is known, processed foods can be consumed and the changes in dietary habits can be expected to improve the QOL of allergy sufferers.

Kondo et al assessed readily available processed egg, milk, and wheat products and measured the protein content of each specific ingredient in each food using ELISA methods, and classified them into 9 concentration levels. A quick reference table for food allergen content was generated^28^^)^. In verification of the content of each specific ingredient in identical products, there was almost no variation even when the year of manufacture differed. On the other hand, in examining identical kinds of foods (e.g., bread and milk), the specific ingredient content differed by up to 100 times depending on the food manufacturer. The ability to identify the amount of safe-to-eat allergen-containing foods (obtained from the results of oral challenge tests) by cross-referencing with the quick reference table is being investigated. The amounts determined to be safe to eat based on the oral challenge test were evaluated in processed food tests. Allergy induction was observed at a rate of 10 to 40% when the processed food contained one-tenth of the safe-to-eat amount. At amounts of one-hundredth or less, the allergy induction rate was 5% or less. Therefore, assuming that the food is consumed at home and that safety is a priority, it is thought that a safety factor of one-hundredth is desirable.

As a precaution, when using the system, even if the product name is identical, the content may differ depending on food manufacturers, thus, it is necessary to confirm the manufacturer’s name and ingredient standards when purchasing. Obligatory or recommended labeling is limited to processed foods, and foods provided by the food service industry, such as fast-food shops, restaurants, and hotels, which are deeply ingrained in social life, are not subject to the labeling system. Information on allergens is provided based on a voluntary initiative by fast food establishments and restaurants. However, these pieces of information are not always checked, and there have been reports of allergy incidents.

## 14. Trends in Quantitative Risk Assessment of Allergy-labeling

In western countries, the concept of minimum incidence model was introduced in 2002 to estimate allergy threshold among population^29^^)^. This initiative was to achieve zero risk for allergic patients, however, its practical application turned out to be extremely difficult due to the very low levels of estimated minimum thresholds amount. Consequently, the use of PAL, which indicates the existence of unintended allergy-inducing ingredients, was more frequently used.

Crevel et al employed the benchmark dose method to quantify the minimum eliciting dose (ED) of protein in allergy-inducing ingredients linked to allergy onset, and considered that the probability of a reaction would be in 1 or 5% of allergy patients at the reference dose. Subsequently, reference doses were published for 11 major allergy-inducing ingredients in collaboration with the Netherlands Organization for Applied Scientific Research (TNO), and the Food Allergy Research and Resource Program (FARRP), a research institute at the University of Nebraska, USA^30^^,^^31^^)^. The Australia-New Zealand Allergen Bureau, an industry group in New Zealand, has adopted a risk assessment method called VITAL, a short for Voluntary Incidental Trace Allergen Labeling, based on this reference dose. Many western food companies and government agencies employ this risk management method. However, other than this initiative, there is still no consensus on a method among European countries, the USA, Canada, Australia, and New Zealand. Subsequently, additional data and methods have been established to develop the use of additional reference doses. Validation studies of ED models using single-dose load tests are also conducted^32^^)^.

In addition, TNO and FARRP collected food challenge test data and developed a model averaging approach. The model averaging approach is considered to be suitable for benchmark value deviations. New ED values ​​for 14 allergy-inducing ingredients were generated. The results were published as VITAL 3.0 values ​​of reference doses of the VITAL program^33^^,^^34^^)^.

In Japan, labeling as a risk management approach has been at the forefront since 2001. In 2016, the Food Safety Commission of Japan (FSCJ) conducted an egg risk assessment, and in 2021, a draft evaluation report was compiled^35^^)^. In the evaluation report, Japan assessed and discussed quantitative evaluation using the ED model, as conducted in western countries. Although the quantitative evaluation is not yet complete, the FSCJ concluded that current labeling system for foods containing allergens is generally appropriate for “eggs”. In the future, it is important to accumulate necessary scientific knowledge in order to carry out food health impact assessment including further refinement^35^^)^.
